# A Systematic Review of Multimodal Frameworks for Assessing Health Vulnerability in Academicians Across Ergonomic, Lifestyle, and Dietary Domains

**DOI:** 10.3390/healthcare14030413

**Published:** 2026-02-06

**Authors:** Pooja Oza, Shraddha Phansalkar, Aayush Shrivastava, Abhishek Sharma, Jun-Jiat Tiang, Wei Hong Lim

**Affiliations:** 1 MIT School of Computing, MIT Art, Design and Technology University, Pune 412201, India; pooja.oza@mituniversity.edu.in (P.O.); shraddha.phansalkar@mituniversity.edu.in (S.P.); 2 Department of CSE, Maharishi Markandeshwar Engineering College, Maharishi Markandeshwar Deemed to be University, Mullana, Ambala 133207, India; mr.aayushshrivastava@gmail.com; 3 Department of Computer Science and Engineering, Graphic Era Deemed to be University, Dehradun 248001, India; abhishek15491@gmail.com; 4 Centre for Wireless Technology, CoE for Intelligent Network, Faculty of Artificial Intelligence & Engineering, Multimedia University, Persiaran Multimedia, Cyberjaya 63100, Selangor, Malaysia; 5 Faculty of Engineering, Technology and Built Environment, UCSI University, Kuala Lumpur 56000, Malaysia; limwh@ucsiuniversity.edu.my

**Keywords:** occupational ergonomics, lifestyle challenges, dietary habits, academicians, teachers, health vulnerability, machine learning, multimodal assessment, taxonomy, NCD (non-communicable diseases)

## Abstract

**Background:** Lifestyle challenges such as prolonged sitting, irregular dietary habits, high stress levels, and lack of physical activity have become increasingly common among working professionals. All these factors contribute to the risk of chronic diseases such as diabetes, heart disease, obesity, and high blood pressure, which in turn result in reduced work performance and quality of life and may further affect health services access through increase healthcare needs. The teaching environment, like many other work environments, is mentally, emotionally, and practically demanding, and it puts extra pressure on those who work in it. Academicians, who devote themselves to guiding young minds, often make unhealthy daily choices and face significant work-related stress, which can lead to serious long-term health problems. This review highlights that health and well-being are shaped not by a single factor such as diet, work patterns, or habits, but by their combined effect. **Methods:** A study of around 113 studies has highlighted that academicians usually feel drained and physically exhausted. **Result:** The factors like prolonged fasts, insufficient water intake, long-standing hours, long and continuous talking, and extended periods in the sitting position have added to their stress levels at the workplace. The most critical finding is that these factors do not affect in isolation but impact as a combined interaction. These issues influence each other, thus increasing the vulnerability to lifestyle disorders. **Conclusions:** This critical problem can be addressed with a Multimodal Assessment Framework that integrates teachers’ data on dietary habits, workplace ergonomics, sleep quality, and levels of physical activity. The presented work also proposes a statistical technique with an Artificial Intelligence (AI) model, and generates Vulnerability Quotient (VQ) that show lifestyle disease-related exposure of the teachers, which may be further used to provide remedial interventions. These insights can further guide institutions and policymakers to design healthier, supportive, and sustainable teaching environments.

## 1. Introduction

Academicians influence society by educating the new generation to drive social, economic, and cultural development. They have a statistically significant effect on increasing educational opportunities and contributing to national economic development, which in turn strengthens the economic stability and improves social welfare of the nation [1,2]. The academicians deliver knowledge and ethical values through teaching and mentorship, while shaping different aspects of culture and society [3,4,5]. Academicians are crucial for society because they influence students to foster moral development, ethical behavior, and nurture the leaders of the next generation. Their role goes beyond knowledge transmission and the promotion of a healthy environment and innovative thinking [6,7].

However, despite their impactful contributions to the emerging professionals, academicians remain deprived of healthy and balanced professional and personal lives [6]. There is a sincere concern of inadequate support, due to limited funding and teaching resources, making it challenging to deliver instruction to the larger student masses with efficiency [8,9,10,11]. Academicians, especially primary teachers, also experience heavy workloads due to teaching responsibilities, mentoring students, and fulfilling administrative duties. All these factors usually result in elevated stress levels and burnout [8]. Their efforts are often undervalued in terms of emoluments and insufficient employer support [12,13,14]. Additionally, the expanded roles of teachers frequently require long working hours devoted to lesson planning, assessment, and participation in extracurricular activities [12,15,16].

The increase in these challenges in a professional life are more vulnerable to lifestyle health disorders like obesity, diabetes, cardiovascular disease, and vocal strain due to occupational stress and physical exhaustion caused by prolonged standing, sustained verbal communication over extended periods and inconsistent or irregular meal patterns [14].

Supporting this concern, different surveys indicate the prevalence of chronic illnesses among teachers, especially for diabetes, obesity, and cardiovascular diseases, as discussed in Table 1:

Thus, it is essential to address the factors that influence teachers’ health, particularly their relationship with lifestyle-related disorders. In light of these findings, most existing studies examine lifestyle factors—such as dietary habits, workplace ergonomics, and health conditions—in isolation. Therefore, an integrated approach is required to predict health vulnerability among teachers by considering the combined interaction of dietary practices, lifestyle challenges, and workplace ergonomics.

To bridge this research gap, this paper proposes a conceptual multimodal assessment approach that holistically integrates these essential components to effectively understand and manage the health risks faced by teachers.

### 1.1. Motivation

Teaching is one of the most academically and emotionally demanding professions. Teachers are expected to function not only as classroom and administrative managers but also as sources of emotional support for their students. These responsibilities are further compounded by occupational demands such as prolonged standing during classroom activities, extensive use of the voice, irregular schedules, sedentary tasks, and sustained emotional stress. Moreover, skipping nutritious meals and facing difficulties in maintaining a healthy lifestyle can gradually impair overall well-being [15,20]. Despite the critical role of teachers’ health in individual development and broader societal well-being, this area remains insufficiently studied.

Teachers face numerous occupational challenges. They perform multiple roles in today’s educational environment—as mentors, counselors, and community leaders—and often work long hours that extend beyond classroom teaching to include lesson planning, grading, and extracurricular responsibilities [12,15]. Prolonged standing, continuous speaking, and irregular meal schedules contribute to occupational stress and physical fatigue, resulting in increasing teachers’ susceptibility to lifestyle-related diseases such as obesity, diabetes, and cardiovascular disorders [21,22], as well as vocal cord strain. Understanding the health problems teachers face and how these relate to lifestyle-related disorders.

### 1.2. Scope of Research

The study of the latest reports [21,22] shows that the academicians suffer from the occurrence of lifestyle disorders like obesity, diabetes, and cardiovascular diseases among teachers. This emphasizes an urgent need for research in this domain. Many educators report physical discomfort, disturbed metabolism, and emotional fatigue and stress resulting from heavy workloads, long-standing hours, and repetitive task pressures [23,24].

Despite these concerns, most of the earlier works on focused occupational groups have been carried out in traditional or static workplace settings and typically focus on assessing a single occupational or lifestyle factor at a time (i.e., unimodal analysis). Very few studies have investigated the complex interactions among the different causal factors, such as lifestyle habits, ergonomics, and dietary pattern on the health quotient of individuals. In the current state of art works, we elicit that there is an urgent need for an integrated, multimodal assessment approach that evaluates these factors simultaneously on the vulnerability analysis of teaching professionals [25].

**Figure 1:**
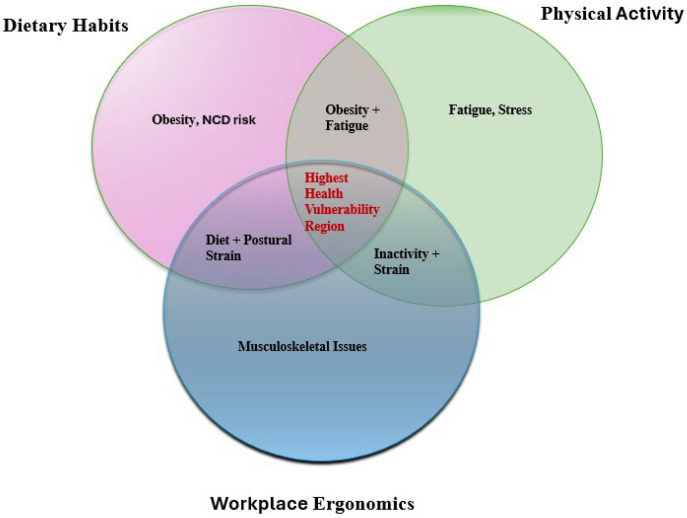
Venn diagram for mapping dietary habits, workplace ergonomics and physical activity interactions, highlighting the highest health vulnerability region.

Three key dimensions—dietary habits, ergonomics, and lifestyle-related disorders—are prioritized and incorporated into a multimodal health assessment approach, as put forth by the earlier studies [26,27,28]. Figure 1 presents a Venn diagram illustrating the intersection among dietary habits, physical activity, and ergonomics and their collective impact on teachers’ health and well-being. The overlapping regions highlight combined risk factors, including obesity, physical inactivity, stress, fatigue, and musculoskeletal strain. The central intersection demonstrates how the highest vulnerability region of the teacher is with poor dietary habits, bad workplace ergonomics and low physical activity.

### 1.3. Objectives

This paper aims to identify health vulnerabilities among teachers by addressing existing research gaps through a comprehensive review of data on dietary practices, lifestyle-related factors, and occupational ergonomics. It emphasizes the relevance of multimodal health monitoring frameworks by finding significant indicators employed across studies to support the early detection and prevention of health risks. The goals are as follows:

1. Identifying key health characteristics and assessment techniques for academicians.
2. Evaluating integrated strategies using multimodal data for the analysis and prediction of academicians’ data.
3. Identify research gaps and propose academicians-focused wellness systems by applying Vulnerability Quotient (VQ) to address individual risks.

**Figure 2:**
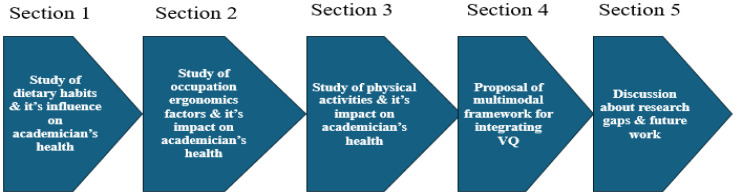
Structure of the paper.

The rest of the paper is structured as follows in Figure 2:

Figure 2 provides the structure of the paper that describes the main goals and flow of the survey. The primary objective of this study is to assess and identify health vulnerabilities among academicians in relation to lifestyle factors, dietary habits, and work ergonomics. The study specifically focuses on the following:

- Systematically review the literature to collect existing evidence about how academicians’ dietary habits, lifestyle behaviors, and workplace ergonomics influence health outcomes.
- Analyze how these factors interact and impact the physical and emotional health as a whole.
- Assess and identify significant gaps in the literature and health assessment methods related to academic health risks, while integrating the Vulnerability Quotient (VQ) to capture the vulnerability of every academician.

### 1.4. Preliminaries

#### 1.4.1. Dietary Habit

Dietary habits refer to the patterns of food consumption that sustain energy, cognitive function, and physical resilience required for sustained workplace performance and stress management [29].

#### 1.4.2. Physical Activity

Physical activity in terms of occupational wellbeing can be defined as work-integrated movements and exercises [30,31] that reduce musculoskeletal pain and improve the efficiency of work.

#### 1.4.3. Occupational Ergonomics

Occupational Ergonomics is the science for developing activities, workplaces, and technology that are specific to employees’ physical and mental capabilities [32,33,34,35] to improve health, safety, and productivity while lowering the risk of work-related injuries.

#### 1.4.4. Multidimensional Health Risks

The convergence of ergonomic stress, unhealthy lifestyles, and poor dietary habits increases vulnerability to health issues, such as:

- Musculoskeletal disorders;
- Chronic fatigue and burnout;
- Anxiety and emotional instability;
- Gastrointestinal and metabolic concerns;
- Reduced teaching performance and job satisfaction.

## 2. Materials and Methods

This review was conducted in accordance with PRISMA 2020 guidelines. The completed PRISMA 2020 Checklist is provided in Supplementary Materials Table S1.

### 2.1. Prisma Diagram

**Figure 3:**
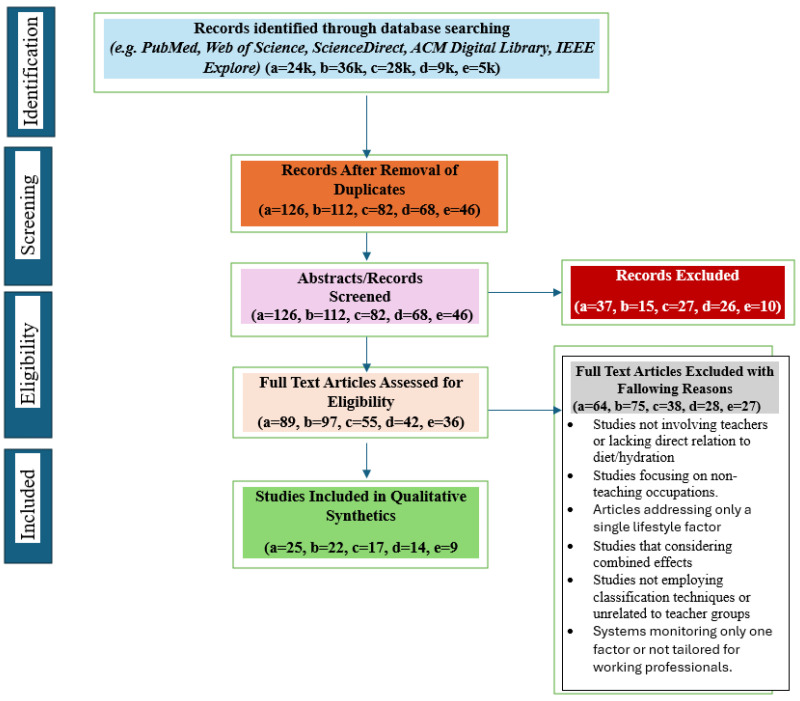
The adopted PRISMA2020 flow diagram for systematic review detailing searches, numbers of abstracts/records, screened and full text articles reviewed where a = impact of lifestyle disorder on professionals health, b = impact of physical activities on teacher’s health, c = impact of occupational ergonomics on teacher’s health, d = impact of dietary habits on teacher’s health, e = multimodality and fusion techniques and on writing reviews.

Figure 3 illustrates the flow diagram as per PRISMA 2020 guidelines, followed to identify, screen, and select relevant studies for this systematic review. During the identification phase, research papers were initially collected from different databases like PubMed, Web of Science, Science Direct, ACM Digital Library, IEEE Explore, etc., which yielded a total of 2.4 k records for category (a), 2.6 k for category (b), 1.8 k for category (c), 1.2 k for category (d), and 1 k for category (e).

Following this, duplicate records were carefully removed, resulting in 126, 112, 82, 68, and 46 unique studies across the respective categories. The titles and abstracts of the remaining articles were examined. At this stage, 37, 15, 27, 26 and 10 of the studies were excluded for not meeting the inclusion criteria. For example, a lack of direct relevance to teachers or educators or incompatibility with research objectives.

During the eligibility stage, we read the full text of 89, 97, 55, 42 and 36 papers. The following reasons led to the exclusion of several papers:

- The study population did not include teachers or was not directly related to dietary or hydration factors.
- The focus was limited to non-teaching or single-factor occupational issues.
- The articles addressed only isolated lifestyle components without examining combined effects.
- The study design lacked classification techniques or was unrelated to teaching contexts.
- The evaluated systems were not integrated or specifically developed for working professionals.

Finally, in the inclusion phase, the studies that met all the predefined eligibility criteria were retained for qualitative synthesis. That led to 25, 22, 17, 14, and 9 papers in each category. Through a systematic and selective review, only 87 studies with high relevance, reliability, and methodological quality were included. These highly selected studies form a strong evidence base on the basis of which the multimodal determinants of health vulnerabilities among academicians can be explored.

### 2.2. Research Questions

The five primary research questions (RQ1–RQ5), which underpin this investigation of academicians’ health and well-being, are presented in Table 2. Each research question focuses on a distinct domain—dietary behaviors, office ergonomics, lifestyle challenges, the interrelationships among these domains, and multimodal assessment approaches. Collectively, these interrelated questions provide a systematic framework for vulnerability assessment and illustrate how the interaction among these factors influences academicians’ overall well-being.

This survey aims to examine the relationship between occupational lifestyle problems, such as food habits, occupational ergonomics, and physical activities, and human health risks, which can lead to obesity, diabetes, and hypertension. Five questions are provided in the table and are addressed in this survey. Lifestyle disorders and their effects on health are included in each Research Question (RQ) in Table 2.

### 2.3. Database-Wise Search String

Databases PubMed, Web of Science, Science Direct, IEEE Xplore, and ACM Digital Libraries are used to retrieve the articles related to specialized search queries related to research. The multiple databases search approach is adopted, as shown in Table 3.

### 2.4. Inclusion and Exclusion Criteria

#### 2.4.1. Study Selection

In Table 4, we have listed the inclusion and exclusion criteria for different categories of searches. The inclusion of studies published from the year 2000 onwards and written in English was justified to ensure that the review captured recent developments in the field while maintaining consistency and clarity in data interpretation.

Study selection and data extraction were conducted independently by two authors. An agreement rate of 80% was observed between the authors for study selection and data extraction. The remaining 20% of discrepancies related to study inclusion or data extraction were resolved through discussion and consensus.

#### 2.4.2. Quality Assessment of Included Studies

The included studies’ methodological quality was assessed using the Newcastle–Ottawa Scale (NOS) for observational studies and the Critical Appraisal Skills Program (CASP) checklist for review studies in Table 5. Studies scoring ≥ 7 were classified as high quality, while scores between 5 and 6 were considered moderate quality.

## 3. Detailed Analysis of Literature Review

### 3.1. RQ1: Influence of Dietary Habits (Eating and Hydration) on Health and Performance of Teaching Professionals

Several studies [36,37,38] have examined the influence of dietary practices on work performance. The evidence from previous research [37,39,40] indicates that the consumption of unhealthy foods, irregular meal timing, and inadequate water intake are associated with metabolic disturbances, reduced energy levels, and impaired cognitive functioning. Insufficient water intake, in particular, has been linked to fatigue, memory loss, and decreased attention span [41,42]. Studies [43,44] further emphasize that maintaining adequate hydration is a critical but generally undermined health habit. This issue is especially relevant for teachers, who generally undergo prolonged teaching and counseling sessions with students, undergoing physical and mental strain requiring both adequate hydration and focus throughout the day.

The findings of studies [43,44,45] indicate that adequate nutrition and proper hydration are significantly associated with improved energy levels, focused delivery, reduced body stress and greater overall job satisfaction. These results support the hypothesis that healthy dietary and hydration habits among academicians lead to reduced vulnerability to lifestyle disorders.

A detailed overview of studies examining dietary behavior patterns among teaching professionals and their association with cognitive, psychological, and occupational health outcomes is provided in Supplementary Materials Table S2 [46,47,48].

The studies show that there are fewer longitudinal and experimental studies specifically examining dietary and water consumption patterns among academicians; most available evidence is cross-sectional and observational in nature. There is also a lack of intervention-based research that highlights a need in the literature, particularly studies that implement and evaluate how changes in eating behaviors impact the health quotient.

### 3.2. RQ2: Impact of Occupational Ergonomic Factors on Academicians’ Well-Being

Teaching is a physically and intellectually demanding profession that involves long sessions of standing, high-pitch vocal stress, repetitive tasks, and often poor workplace ergonomics. Ergonomic challenges in academic settings lead to musculoskeletal discomfort, vocal strain, and mental fatigue [49,50,51]. Academicians frequently report pain in the neck, back, and shoulders due to extended teaching sessions and inadequate classroom ergonomics [51,52]. Long working hours and emotional interactions with the students further amplify these challenges by reducing concentration and increasing stress levels [53]. Consequently, several studies emphasize that the implementation of appropriate ergonomic interventions is essential to reduce these occupational health risks.

Additionally, online and digital teaching practices have become common in education, and studies highlight the impact of prolonged screen time on the teacher’s vulnerability to health risks [54]. Similarly, there is a lack of studies that investigate how schools and educational institutions handle the ergonomic challenges through institutional policies, guidelines, or support mechanisms.

Supplementary Materials Table S3 summarizes major studies on occupational ergonomics among teaching professionals, highlighting the impact of posture, working hours, and interventions on physical discomfort and fatigue levels.

Prolonged standing in secondary school teachers who stand for more than four hours per day have a higher risk of developing knee and lower back problems [49]. Hoarseness is another frequently reported issue, particularly among teachers working in primary grades. A clinical survey conducted in India found that approximately 65% of teachers reported symptoms such as fatigue, vocal strain and general physical discomfort [50,52].

Classroom design, poorly designed desks and chairs and furniture quality often affect physical health and are associated with a higher prevalence of musculoskeletal discomfort, particularly among university teachers [55]. Studies show that academicians who work more than fifty hours per week are more susceptible to mental fatigue and burnout, which adversely affect both personal well-being and overall performance [56]. The study highlights significant research gaps and establish need for further investigation.

For instance, although digital education has been established as the new norm within many educational institutes, there is a lack of studies on the impact of prolonged screen time in classroom settings [54]. Most existing studies examine occupational health issues—such as posture-related strain or vocal fatigue—in isolation, without study of their interactions. Similarly, the long-term consequences of increased screen exposure due to digital teaching remain largely unexplored [54].

However, besides fewer studies examining physical symptoms—such as back pain—there is a lack of experiments and data addressing the mental health consequences of occupational demands among academicians. This gap highlights the need for a more in-depth understanding of how multiple work-related factors, including physical and ergonomic factors, interact to influence the overall well-being of academicians. Addressing these underexplored areas is essential for developing effective strategies that support both the physical and psychological health of educators in contemporary exosystems.

### 3.3. RQ3: Lifestyle Challenges and Overall Wellness of Academician

In today’s time-constrained, technology-savvy environment, excessive screen time and limited physical activity lead to bad effects on the health of working professionals, including those in the education sector. Academicians are especially vulnerable due to high levels of work-related stress, heavy workloads of grading responsibilities, and the use of digital technologies for teaching and academic tasks.

Inadequate sleep may impair cognitive–emotional functioning and immune capacity, both of which are essential for effective teaching [57]. Excessive screen time has been associated with higher anxiety levels and poor sleep quality [54]. Conversely, insufficient physical activity can lead to fatigue and adversely affect cardiovascular health as well as mental well-being [58]. Many academicians also lack awareness of appropriate sleep hygiene practices, further intensifying these challenges [59]. Additionally, sedentary behaviors, such as prolonged sitting, have been linked to increased metabolic risks [60].

The overview of the literature on lifestyle challenges and teacher wellness provided in Supplementary Material Table S4 [61] compiles major studies examining lifestyle-related determinants—such as sleep, screen exposure, and physical activity—affecting the psychological and physical well-being of educators across various teaching levels.

### 3.4. RQ4: Interactions Among Diet, Ergonomics, and Lifestyle in the Teaching Profession

Far fewer studies have examined the combined effects of dietary practices, ergonomic environments, and lifestyle challenges compared with the extensive literature assessing individual factors. For example, poor dietary habits may aggravate the adverse effects of prolonged working hours or lack of sleep. Academicians are frequently exposed to multiple, interrelated occupational impacts, highlighting the need for a more integrated approach to their health and well-being.

Recent findings suggest the need for a multivariate approach. Regular physical activity, improved sleep quality, and a healthy diet have been associated with reduced stress levels and increased job satisfaction [62,63]. Additionally, studies have demonstrated that screen time, ergonomic conditions, and hydration habits are significant predictors of fatigue and exhaustion in teachers [64].

Studies exploring multidimensional health factors in academicians, provided in Supplementary Materials Table S5, highlight empirical studies that examined the combined effects of dietary habits, ergonomics, physical activity, and other lifestyle parameters on the occupational well-being of academicians, emphasizing the need for integrated multimodal wellness frameworks.

### 3.5. RQ5: Key Components of an Effective Multimodal Monitoring Solution

Advances in healthcare and intelligent systems have led to the development of multimodal systems that monitor human well-being through the integration of multiple data sources and formats. These systems analyze significant key health factors, including diet, hydration, sleep, physical activity, and ergonomic behavior.

For example, the Fit Work Edu system integrates ergonomic monitoring with hydration tracking and has been established to improve back pain outcomes and hydration levels [65]. Studies have also demonstrated the effectiveness of wearable and mobile health devices in increasing daily water intake and physical activity, such as step counts, active energy levels, etc. [66]. Additionally, platforms such as LIFEMAP enhance behavioral awareness of users by calibrating stress indicators and sleep-related parameters [67]. AI-driven systems employing image recognition and motion-sensing technologies further improve effective dietary monitoring and posture tracking, as presented in recent research [68].

Table S6, provided in the Supplementary Materials, discusses studies or systems, highlighting features and outcomes of such health monitoring technologies and summarizing representative multimodal systems and digital platforms integrating dietary activity, ergonomic, and behavioral components for health monitoring. Reported outcomes emphasize improvements in hydration, activity adherence, and stress reduction among varied user groups.

## 4. Discussion and Gaps

RQ1 research study [69,70,71] indicates that poor dietary habits lead to increased fatigue, stress and memory impairment. In contrast, studies [72,73,74] report that regular dietary habits and water intake lead to positive mood, reduced anxiety levels, and greater mindfulness in the classroom. These findings highlight the significance of wellness programs at workplace that create awareness of healthy dietary practices and adequate hydration among academicians. In studies related to RQ2, studies show that teaching involves prolonged standing, continuous speaking, and extended working hours, often within ergonomically substandard environments. These occupational demands contribute to physical health issues such as musculoskeletal pain [20,49,55], vocal stress [52,75], and mental fatigue [53,76,77,78]. Intervention studies demonstrate that ergonomic improvements—such as the provision of comfortable seating, periodic breaks, and recreation activities—can significantly reduce physical stress and enhance overall well-being [56]. RQ3 discussed several studies that report that poor sleep quality [57,79] and excessive screen time [72] are common issues among academicians. Longer screen times are associated with increased stress and disturbed sleep, which in turn adversely affect emotional well-being and mental concentration. Additionally, extended periods of sitting and physical inactivity contribute to fatigue, health hazards, and reduced job satisfaction [60]. In RQ4, a study by the researchers [59] suggests that academicians who maintain a healthy diet, obtain adequate sleep, and engage in regular physical activity experience lower stress levels and greater job satisfaction. Other studies further indicate that excessive screen use, poor posture, and dehydration significantly contribute to exhaustion and burnout [31]. Moreover, studies [80,81] demonstrate that interventions integrating lifestyle modification, health awareness, and workplace improvements are more effective than studies of impacts targeting individual factors. RQ5 suggested the need for multimodal health monitoring systems to provide systems with integrating data from multiple sources, including nutrition, hydration, sleep patterns, physical activity, and posture. Studies [66,67,68,82] discuss the use of wearable devices, healthcare applications, and structured health programs to support health monitoring. Collectively, these systems would promote an understanding of the health profile of an individual and detect health-related risks among academicians.

Based on the review of the literature corresponding to RQ1–RQ5, academicians’ well-being has predominantly been examined through short-term studies and single-factor approaches (e.g., diet, posture, or stress), with limited consideration of how these factors interact and collectively influence daily teaching practices. Critical issues such as data privacy, accessibility to health-monitoring tools, and the representation of academicians from rural or resource-constrained settings are often overlooked. Furthermore, mental health is rarely examined in conjunction with physical and occupational factors. The absence of integrated, multimodal frameworks and clear policy direction highlights the need for such approaches and systems to effectively safeguard the well-being of the teaching professionals.

## 5. Proposed Architecture

**Figure 4:**
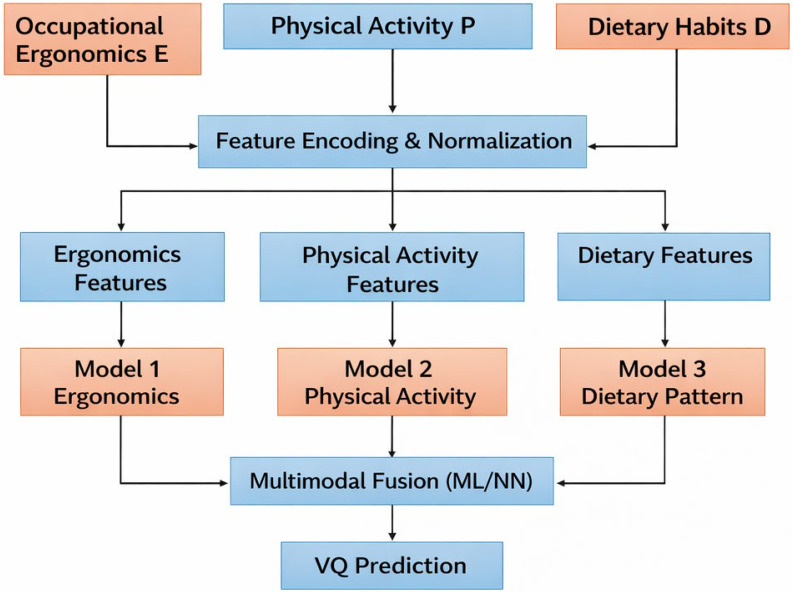
Vulnerability Quotient (VQ) prediction framework.

The Vulnerability Quotient (VQ) is a predictive index derived through feature selection and neural network-based analysis of multiple input variables, including ergonomic factors, physical activity, and dietary habits. This composite score reflects the potential health risk of individual teachers, thereby supporting the early identification of health concerns and informing preventive strategies aimed at taking care of overall well-being. As illustrated in Figure 4, the multimodal framework employed to assess health vulnerability (VQ) [83,84] among teaching professionals evaluates three key domains:

The proposed conceptual VQ framework, illustrated in Figure 4, represents a comprehensive health assessment model that integrates three essential domains: occupational ergonomics, physical activity, and dietary habits. This conceptual model will be developed by following a systematic review of studies related to occupational ergonomics, physical activity, and dietary habits. The VQ calculation, as presented in this conceptual framework, serves as a preliminary roadmap to guide future empirical model development and validation.

### 5.1. Conceptual Model Inputs

The VQ-based conceptual model interprets health vulnerability as the cumulative effect of three core domains:

1. Ergonomics (E)

This domain evaluates posture, workstation design, and screen-related ergonomic factors. Habitual practices such as prolonged sitting, repetitive neck flexion, and working at poorly designed workstations can contribute to musculoskeletal strain, persistent fatigue, and recurrent pain [85]. Data for this domain will be derived from peer-reviewed literature and validated questionnaires.

2. Physical Activity (P)

This domain incorporates variables such as time spent sitting, number of steps taken, overall physical activity level, sleep quality, and screen time. Insufficient physical activity and poor sleep quality are widely associated with increased health risks, including obesity, cardiovascular disease, and metabolic disorders [86]. Data for this domain will be obtained from established physical activity assessment tools and relevant empirical studies.

3. Dietary Habits (D)

This region is involved in the quality of diet, level of hydration, calorie balance and consumption of processed foods. Factors that contribute to causing problems may be skipped meals, low intake of water, and unhealthy eating habits. Processed food is also a usual cause for diabetes, obesity or heart ailments [87]. In this conceptual framework, dietary data is assumed to be collected from validated dietary assessment tools.

The resulting values of the conceptual model will be finally normalized to the same low level in order to be able to compare directly across different scales, like hours, calories, and frequency.

### 5.2. Conceptual Model Feature Extraction and Normalization

Feature extraction allows raw data from different domains to be transformed into a standardized form. In this stage, the heterogeneous inputs like hours, frequencies, heartbeats, etc., will be represented on a comparable scale. Here is the mathematical representation of our conceptual framework, as follows:

Let $X_{ij}$ indicate the raw value of $j^{th}$ lifestyle factor for $i^{th}$ academician, where the indicator belongs to one of three domain occupational ergonomics, physical activity, and dietary habits.

$$f_{ij}=\frac{x_{ij-\min\left(x_{j}\right)}}{\max\left(x_{j}\right)-min\;(x_{j})}$$

where $f_{ij}$ = normalized feature corresponds to lifestyle factor *j*. After feature extraction, the resulting feature vectors for each domain are denoted as $F_{E}$, $F_{P}$, and $F_{D}$ respectively.

### 5.3. Domain-Specific Conceptual Models

Domain-specific sub-models are illustrated to emphasize that each lifestyle dimension contributes uniquely to vulnerability assessment. These sub-models are not implemented or tested in the present study; rather, they represent conceptual abstractions derived from patterns observed across the reviewed literature. Each domain is proposed independently using a domain-specific analytical model:

Model 1: Ergonomics-based vulnerability towards lifestyle disorders: $V_{E\;}=g_{E\;}\;(F_{E})$.
Model 2: Physical activity and sleep-based vulnerability: $V_{P}=\;g_{P}\;(F_{P})$.
Model 3: Diet-based vulnerability: $V_{D\;}=\;g_{D}\;(F_{D})$.

Where $F_{E,\;}\;F_{P,}\;F_{D}$ are sets of extracted features for domains occupational ergonomics, physical activity, dietary habits, respectively, and $g_{E\left(.\right)},\;g_{P\left(.\right)}$, $g_{D(.)}$ are domain-specific analytical functions (statistical or ML-based).

### 5.4. Multimodal Feature Fusion

Multimodal feature aggregation is a process where outputs from individual domains will be conceptually combined to estimate an overall Vulnerability Quotient (VQ).

This aggregation is presented as a theoretical mechanism to illustrate how multidimensional lifestyle risks could be integrated in future analytical or machine learning-based implementations.

Conceptual multimodal fusion:

$$VQ=w_{E\;}V_{E}\;+\;w_{P}V_{P}\;+\;w_{D}V_{D}$$

where

- VQ = Vulnerability Quotient;
- $V_{E},V_{P},V_{D}$ = Unimodal vulnerability scores;
- $w_{E},w_{P},w_{D}$ = Weighting coefficients reflecting relative contribution.

Alternatively, the fusion process may be expressed in a generalized form, as follows:

$$VQ=h(V_{E},V_{P},V_{D})$$

where $h\left(.\right)$ represents a generic multimodal fusion function.

The proposed conceptual *VQ* framework does not represent an empirically validated model and should not be interpreted as an evidence-tested outcome of the review. Instead, it serves as a conceptual synthesis and a future research roadmap, translating fragmented evidence from the literature into a coherent structure that can guide empirical validation using real-world and longitudinal datasets in subsequent studies.

## 6. Limitations of Work

This systematic literature review is limited by keyword used while selecting the literature. Academicians’ health and wellness is a specific domain for teachers; we found fewer studies related to teachers. Hence, the scope of the study was broadened to include other occupations. Only 87 studies were selected for the survey, including the grey literature like analytical reports, websites from 2000 to 2025. Manual screening of studies was conducted, which were obtained from various databases like PubMed, ACM, IEEE, etc. This review was limited to popular techniques, such as AI, ML, feature extraction and fusion techniques. Thus survey suffers from the threat of bias due to the search query used. This paper shows the conceptualization of the proposed model for Vulnerability Quotient detection as an alternative to the studied solutions in the literature with respect to a variety of datasets, and a lack of validation. The proposed architecture is under experimentation and evaluation is not stated in this paper.

This multimodal framework provides a useful lens through which to consider academicians’ health risks; however, it does have definite limitations. Since the model relies heavily on self-reported and observational data, there is the potential for bias or error in the measure. Furthermore, the weighting for the prioritization of risks and the choice of other features came from expert judgment, which is valuable, but both decisions should be tested with larger and more diverse datasets if the findings are to have more certainty in generalizability.

Additionally, the framework is mainly focused on physical health risks associated with academicians—including ergonomics, physical activity, and dietary status—as psychosocial factors (e.g., work-related stress, emotional status, and organizational climate) were not explicitly part of formal consideration using the framework, despite the effect of these on teacher health being known. Further, the framework does not account for time, providing only a static, cross-sectional purpose; being temporal would allow it to capture the risk factors over time. Also, multiple domains will have to be analyzed, which may be harder to implement in relatively low-resourced educational contexts. The model has not yet been fully applied or validated as a real-world model.

These limitations highlight the necessity for future studies to improve data collection methods, broaden health factors, and systematically assess reliability and predictive capacity across various academicians.

## 7. Conclusions and Future Work

The purpose of this study is to highlight research gaps in occupational health and well-being of teaching professions with an integrated and multimodal framework. Specifically, multimodality in preventive healthcare of teaching professionals needs integration of multiple types of data of varied factors impacting the health of the professional. In the present work, a thorough study of the effects of dietary habits, workplace ergonomics, and lifestyle practices on the health and performance of teaching professionals was conducted. Through a structured review of the literature (RQ1–RQ3), it was highlighted that poor nutrition, less hydration, prolonged standing, vocal strain, and sedentary lifestyle contributed significantly to both physical and mental fatigue in teaching professionals. These risk factors have been seen impacting in the form of increased stress, musculoskeletal issues, emotional spikes, and reduced job satisfaction. The combined study of these domains examined in RQ4 suggests that lifestyle, dietary, and ergonomic factors do not act independently, but have interaction effects. A strong multimodal monitoring framework proposed in RQ5 aims to design and implement an integrated system that tracks dietary habits, physical activity, ergonomics, and sleep patterns. The presented statistical model is a first step towards the development of a multimodal integrated Vulnerability Quotient predictor for a teaching professional that shows their risk degree to lifestyle disorders.

Future studies are needed to carry out long-term investigations to find out how eating, ergonomics, and lifestyle variables affect the health of the academic community. Further studies should also expand predictive models to include mental health, including mental ergonomics, to better understand their impact on occupational vulnerability and lifestyle-related disorders within the proposed framework. There is scope to extend this work for professions other than teaching to enhance well-being for the general population. It would also be beneficial to include Vulnerability Quotient-predicting AI systems in workplace ecosystems for timely interventions. Future work will focus on realizing and validating the proposed VQ framework through primary data collection and model testing across different occupational groups.

## Figures and Tables

**Table 1:** Summary of prevalent lifestyle disorders among educators in different countries.

Condition	Country/Region	Findings	References
Diabetes	Malaysia	4.1% diagnosed; 5.1% undiagnosed; 5.6% impaired fasting glucose	[17]
	India (Bengaluru)	4.4% diagnosed; 20.7% high risk; 6.2% high blood sugar	
	India (Urban Centers)	14.6% total (6.5% known, 8.1% new)	
Obesity	United States	77% overweight/obese; exceeds national female average	[18]
	Brazil	47.2% overweight/obese; higher among men	
	India (Female Teachers)	70.2% obese (43.2% grade I, 20.4% grade II, 6.6% grade III)	
Cardio Vascular Disease(CVD)	Poland	4.6% of education employees affected; ~30% reported chronic diseases	[19]
	Germany (Mainz Region)	31.7 cases per 10,000 person-years (5-year incidence)	

CVD = cardiovascular disease. Data extracted from national and regional cohort and cross-sectional studies among educators and similar occupational groups.

**Table 2:** Research questions and corresponding discussion points.

RQ No.	Research Question	Discussion Focus
RQ1	How do dietary habits (eating and hydration) influence the health and performance of teaching professionals?	Explores the relationship between self-reported well-being and performance of academicians and their eating and hydration habits, focusing on how dietary choices are perceived to affect daily work and productivity.
RQ2	What is the impact of occupational ergonomic factors (working hours, physical activity, and vocal workload) on academicians’ well-being?	Examines the ergonomic challenges faced by academicians in daily routines and measures the impacts of factors such as working hours, physical exertion, and voice use on physical and mental health outcomes.
RQ3	How do lifestyle challenges (sleep quality, screen time, and physical fitness) affect the overall wellness of academicians?	Discusses how academicians perceive and manage lifestyle factors, including sleep, screen time, and exercise, emphasizing their influence on job satisfaction, motivation, and stress.
RQ4	What are the relationships and interactions among dietary habits, ergonomic stressors, and lifestyle challenges in the teaching profession?	Identifies integrated patterns among diet, ergonomics, and lifestyle data to understand how these dimensions interact and collectively affect academicians’ professional and personal well-being.
RQ5	What are the key components and features of an effective multimodal solution for monitoring diet plan, physical activity, and occupational interfering factors?	Focuses on understanding user requirements, evaluating existing interventions, and testing the performance of integrated multimodal systems designed to enhance academicians’ well-being and monitor risk factors.

RQ = Research Question. Each question aligns with the thematic domains of dietary habits, ergonomics, and lifestyle challenges, serving as the foundation for developing a multimodal well-being assessment framework.

**Table 3:** Database-wise search strategy.

Sr. No.	Database	Search String
1	PubMed	(“dietary patterns” OR diet OR hydration OR “eating behaviour”) AND (“lifestyle factors” OR “lifestyle challenges”) AND (“ergonomic factors” OR “occupational ergonomics” OR “musculoskeletal strain”) AND (“working hours” OR “physical activity” OR “screen time” OR sleep OR “vocal workload”) AND (teachers OR educators OR “teaching professionals” OR instructors) AND (“occupational stress” OR burnout OR wellbeing OR “mental health” OR “occupational health”)
2	ScienceDirect	(“dietary patterns” OR diet OR hydration OR “eating behavior”) AND (“lifestyle factors” OR “lifestyle challenges”) AND (“ergonomic factors” OR “occupational ergonomics” OR “musculoskeletal strain”) AND (“working hours” OR “physical activity” OR “screen time” OR sleep OR “vocal workload”) AND (teachers OR educators OR “teaching professionals” OR instructors) AND (“occupational stress” OR burnout OR wellbeing OR “mental health” OR “occupational health”)
3	Web of Science	TS = (“dietary patterns” OR diet OR hydration OR “eating behaviour”) AND TS = (“lifestyle factors” OR “lifestyle challenges”) AND TS = (“ergonomic factors” OR “occupational ergonomics” OR “musculoskeletal strain”) AND TS = (“working hours” OR “physical activity” OR “screen time” OR sleep OR “vocal workload”) AND TS = (teachers OR educators OR “teaching professionals” OR instructors) AND TS = (“occupational stress” OR burnout OR wellbeing OR “mental health” OR “occupational health”)
4	IEEE Xplore	(“multimodal system” OR “integrated solution” OR “digital health platform”) AND (“diet monitoring” OR “diet plan tracking”) AND (“physical activity monitoring” OR “fitness tracking”) AND (“occupational factors” OR “work-related interference”)
5	ACM Digital Library	(“multimodal system” OR “digital health platform”) AND (“diet monitoring” OR “physical activity monitoring”) AND (“occupational factors” OR teachers)

**Table 4:** Research Question-wise inclusion and exclusion criteria.

Sr. No.	Research Question	Inclusion Criteria	Exclusion Criteria	Measurable Outcomes
1	RQ1	Teachers’ nutrition, hydration, and their impact on physical/mental performance	Non-teachers or lacking direct relation to diet/hydration	Correlation between dietary habits and health/performance indicators (e.g., energy levels, absenteeism, cognitive performance)
2	RQ2	Ergonomic stressors in teaching (e.g., posture, work hours, vocal strain)	Non-teaching occupations or limited to a single factor	Measurable impact on well-being parameters such as fatigue, stress, and posture-related issues
3	RQ3	Lifestyle habits and wellness among educators or similar professions	Articles lacking empirical data or addressing only a single lifestyle factor	Composite wellness indicators related to lifestyle habits, including stress, physical health, and satisfaction
4	RQ4	Combining two or more factors among teacher populations	Only one dimension without considering combined effects	Cross-factor analysis highlighting significant interactions and overlapping risk patterns
5	RQ5	Health monitoring systems involving multiple lifestyle domains (diet, activity, occupation)	Only one factor or not tailored for working professionals	Design blueprint or feature matrix of multimodal health monitoring tools, including feedback, data collection, and personalization capabilities

**Table 5:** Grouped summary of quality appraisal studies.

Study Category	Study Design	Appraisal Tool	Overall Quality	Number of Studies
Teacher-based observational studies	Cross-sectional/Observational	Newcastle–Ottawa Scale (NOS)	High	11
Teacher-based observational studies	Cross-sectional/Observational	Newcastle–Ottawa Scale (NOS)	Moderate	8
Longitudinal and cohort studies (teachers)	Longitudinal/Cohort	Newcastle–Ottawa Scale (NOS)	High	2
Review-based studies (teacher-focused)	Narrative/Systematic/Scoping review	CASP	High	2
Review-based studies (teacher-focused)	Narrative/Systematic/Scoping review	CASP	Moderate	1

## Data Availability

No new data were created or analyzed in this study. Data sharing is not applicable to this article.

## Supplementary Materials

The following supporting information can be downloaded at: https://www.mdpi.com/article/10.3390/healthcare14030413/s1.
