# Supplementary material for: A Systematic Review of Multimodal Frameworks for Assessing Health Vulnerability in Academicians Across Ergonomic, Lifestyle, and Dietary Domains
Source: Healthcare (Basel). 2026 Feb 6;14(3):413. doi: 10.3390/healthcare14030413 (PMC12897325; doi:10.3390/healthcare14030413)
Supplement: Supplementary file 1 [file healthcare-14-00413-s001.zip › healthcare-4054397-supplementary.pdf]

Table S1: PRISMA Checklist

Section and Topic	Item #	Checklist item	Location where item is reported
**TITLE**			
Title	1	Identify the report as a systematic review.	Line No 2
**ABSTRACT**			
Abstract	2	See the PRISMA 2020 for Abstracts checklist.	Line no 18
**INTRODUCTION**			
Rationale	3	Describe the rationale for the review in the context of existing knowledge.	Line no 111
Objectives	4	Provide an explicit statement of the objective(s) or question(s) the review addresses.	Line no 137/225
**METHODS**			
Eligibility criteria	5	Specify the inclusion and exclusion criteria for the review and how studies were grouped for the syntheses.	Line no 260
Information sources	6	Specify all databases, registers, websites, organisations, reference lists and other sources searched or consulted to identify studies. Specify the date when each source was last searched or consulted.	Line no 241
Search strategy	7	Present the full search strategies for all databases, registers and websites, including any filters and limits used.	Line no 245
Selection process	8	Specify the methods used to decide whether a study met the inclusion criteria of the review, including how many reviewers screened each record and each report retrieved, whether they worked independently, and if applicable, details of automation tools used in the process.	Line no 219
Data collection process	9	Specify the methods used to collect data from reports, including how many reviewers collected data from each report, whether they worked independently, any processes for obtaining or confirming data from study investigators, and if applicable, details of automation tools used in the process.	Line no 251
Data items	10a	List and define all outcomes for which data were sought. Specify whether all results that were compatible with each outcome domain in each study were sought (e.g. for all measures, time points, analyses), and if not, the methods	NA

Section and Topic	Item #	Checklist item	Location where item is reported
		used to decide which results to collect.	
	10b	List and define all other variables for which data were sought (e.g. participant and intervention characteristics, funding sources). Describe any assumptions made about any missing or unclear information.	NA
Study risk of bias assessment	11	Specify the methods used to assess risk of bias in the included studies, including details of the tool(s) used, how many reviewers assessed each study and whether they worked independently, and if applicable, details of automation tools used in the process.	Line no 283/527
Effect measures	12	Specify for each outcome the effect measure(s) (e.g. risk ratio, mean difference) used in the synthesis or presentation of results.	NA
Synthesis methods	13a	Describe the processes used to decide which studies were eligible for each synthesis (e.g. tabulating the study intervention characteristics and comparing against the planned groups for each synthesis (item #5)).	NA
	13b	Describe any methods required to prepare the data for presentation or synthesis, such as handling of missing summary statistics, or data conversions.	NA
	13c	Describe any methods used to tabulate or visually display results of individual studies and syntheses.	NA
	13d	Describe any methods used to synthesize results and provide a rationale for the choice(s). If meta-analysis was performed, describe the model(s), method(s) to identify the presence and extent of statistical heterogeneity, and software package(s) used.	NA
	13e	Describe any methods used to explore possible causes of heterogeneity among study results (e.g. subgroup analysis, meta-regression).	NA
	13f	Describe any sensitivity analyses conducted to assess robustness of the synthesized results.	NA
Reporting bias assessment	14	Describe any methods used to assess risk of bias due to missing results in a synthesis (arising from reporting biases).	NA

Section and Topic	Item #	Checklist item	Location where item is reported
Certainty assessment	15	Describe any methods used to assess certainty (or confidence) in the body of evidence for an outcome.	NA
**RESULTS**			
Study selection	16a	Describe the results of the search and selection process, from the number of records identified in the search to the number of studies included in the review, ideally using a flow diagram.	NA
	16b	Cite studies that might appear to meet the inclusion criteria, but which were excluded, and explain why they were excluded.	NA
Study characteristics	17	Cite each included study and present its characteristics.	NA
Risk of bias in studies	18	Present assessments of risk of bias for each included study.	NA
Results of individual studies	19	For all outcomes, present, for each study: (a) summary statistics for each group (where appropriate) and (b) an effect estimate and its precision (e.g. confidence/credible interval), ideally using structured tables or plots.	NA
Results of syntheses	20a	For each synthesis, briefly summarise the characteristics and risk of bias among contributing studies.	NA
	20b	Present results of all statistical syntheses conducted. If meta-analysis was done, present for each the summary estimate and its precision (e.g. confidence/credible interval) and measures of statistical heterogeneity. If comparing groups, describe the direction of the effect.	NA
	20c	Present results of all investigations of possible causes of heterogeneity among study results.	NA
	20d	Present results of all sensitivity analyses conducted to assess the robustness of the synthesized results.	NA
Reporting biases	21	Present assessments of risk of bias due to missing results (arising from reporting biases) for each synthesis assessed.	NA
Certainty of evidence	22	Present assessments of certainty (or confidence) in the body of evidence for each outcome assessed.	NA
**DISCUSSION**			

Section and Topic	Item #	Checklist item	Location where item is reported
Discussion	23a	Provide a general interpretation of the results in the context of other evidence.	NA
	23b	Discuss any limitations of the evidence included in the review.	Line no 528
	23c	Discuss any limitations of the review processes used.	Line no 528
	23d	Discuss implications of the results for practice, policy, and future research.	NA
**OTHER INFORMATION**			
Registration and protocol	24a	Provide registration information for the review, including register name and registration number, or state that the review was not registered.	Not Registered
	24b	Indicate where the review protocol can be accessed, or state that a protocol was not prepared.	NA
	24c	Describe and explain any amendments to information provided at registration or in the protocol.	NA
Support	25	Describe sources of financial or non-financial support for the review, and the role of the funders or sponsors in the review.	Line no 598
Competing interests	26	Declare any competing interests of review authors.	Line no 599
Availability of data, code and other materials	27	Report which of the following are publicly available and where they can be found: template data collection forms; data extracted from included studies; data used for all analyses; analytic code; any other materials used in the review.	NA

Table S2 : Overview of literature on dietary habits, hydration, and academician performance:

Study / Author	Focus Area	Key Findings	Sample Population	Research Method
[46], [47], [48], [49]	Breakfast skipping and concentration in teachers	• Skipping morning meal<br>• Elevated levels of fatigue<br>• Diminished cognitive acuity<br>• Increase obesity.	Kindergarten to 12th-grade teachers, USA, university teachers from Hungary	Cross-sectional survey
[50]	Water intake and mental clarity	• Dehydration<br>• Reductions in cognitive functions	older Spanish population	Experimental
[51]	Diet quality and occupational stress	• High sugar<br>• Low fiber intake<br>• Increased occupational stress<br>• and absenteeism.	Women of reproductive age(UK)	Cross sectional study
[52]	Eating frequency and emotional stability	• Regular meal patterns<br>• Better mood regulation<br>• Lower anxiety levels	Secondary teachers, Brazil	Mixed-methods
[53], [54]	Caffeine and hydration practices	• Caffeinated beverages<br>• Sleep disturbances<br>• Heightened irritability	College instructors	Observational

Table S3 : Overview of literature on ergonomic stressors and academician well-being:

**Study / Author**	**Focus Area**	**Key Finding**	**Population**	**Method**
[20], [55]	Standing posture and musculoskeletal pain	• Prolonged standing per day<br>• Increased reports of back and knee discomfort	High school teachers, UK	Cross-sectional
[56], [58],[62]	Vocal load and voice disorders	• Symptoms indicative of vocal strain or hoarseness	Elementary teachers, India	Clinical survey
[63]	Classroom ergonomics	• Ineffectively designed workstations with elevated discomfort ratings and postural complaints	University lecturers, Spain	Ergonomic assessment
[59], [64], [65]	Working hours and fatigue	• More Working hours<br>• Higher levels of mental fatigue	Secondary school educators	Longitudinal study
[60]	Ergonomic intervention outcomes	• Implementation of ergonomic seating structured breaks reduced musculoskeletal pain and improved reported comfort levels	Middle school teachers	Pilot intervention

Table S4 : Overview of literature on lifestyle challenges and teacher wellness:

**Study / Author**	**Focus Area**	**Key Findings**	**Population**	**Method**
[66], [70]	Sleep quality and burnout	• Poor sleep quality<br>• Elevated levels of burnout<br>• Emotional exhaustion.	Public school teachers, United States	Survey-based
[61]	Screen time and mental health	• Excessive digital screen exposure<br>• Increased anxiety levels<br>• Reduced attention span.	Online teaching staff, Korea	Cross-sectional
[67]	Physical fitness and life satisfaction	• Participation in physical activity<br>• Enhanced life satisfaction<br>• Reduced fatigue	Primary school teachers	Quantitative
[68], [71]	Sleep hygiene awareness	• Insufficient awareness and implementation of healthy sleep hygiene	Secondary school educators	Qualitative
[69]	Sedentary behaviour	• Extended sitting durations<br>• Higher metabolic risk<br>• Decreased job satisfaction	University faculty	Observational

Table S5 : Studies exploring multidimensional health factors in academicians:

Study / Author	Factors Explored	Key Findings	Method	Population
[72]	Diet + Physical activity + Sleep	• healthy lifestyle behaviors<br>• Improved job satisfaction<br>• Reduced stress levels	Structural equation modelling	Teaching faculty, Asia
[34][73]	Ergonomics + Leisure activities + Physical activities	• Work-related stress<br>• Ergonomic strain<br>• Insufficient leisure<br>• Low physical activity	Regression analysis	School teachers, India
[74]	Lifestyle + Occupational stress	• Stress resulting from prolonged working hours	Longitudin al	German educators
[75]	Nutrition + Sleep + Workload	• Unhealthy lifestyle patterns<br>• Poor sleep contributed to higher workplace stress and burnout	Mixed methods	Urban teachers, India
[76]	Integrated wellness factors	• Comprehensive wellness programs improving mental health than isolated single-factor interventions.	Interventio n study	Latin American teachers

Table S6: Features of health monitoring systems:

Study / System	Components Included	Key Features	Target Users	Outcomes Reported
[77]	Diet, hydration, physical activity	• Real-time alerts<br>• Weekly reports<br>• Ergonomic guidance	Teachers and trainers	Improved hydration and reduced back pain
[78]	Mobile + wearable-based monitoring	• Smartphone app and<br>• Smartwatch synchronization for holistic wellness tracking	Working professionals	Increase in daily step count and water intake
[79]	Lifestyle and stress tracking	• Monitoring of sleep<br>• Screen time<br>• Nutrition, and<br>• Stress levels	Nursing home residents	Reported reduction in stress and improved sleep quality
[80]	AI-based dietary intake and calorie counting	• Image-based meal recognition for automated calorie estimation	Patients with type 2 diabetes	Higher compliance with diet logging and nutritional awareness
[81]	Nutrition tracking and diet-planning	• Tracks nutritional intake<br>• personalized diet plans	General population	Provides actionable insights into eating habits

application	and nutrient balance
